# Enhancing the validity of virucidal activity testing of chemical disinfectants by establishing new reference substances according to EN 14476:2019

**DOI:** 10.3205/dgkh000579

**Published:** 2025-09-09

**Authors:** Kira-Marie Roesch, Claudia Hildebrandt, Maren Eggers, Florian Brill, Michele Cavalleri, Jürgen Gebel, Birgit Hunsinger, Sophie Loeffert, Marvin Rausch, Ingeborg Schwebke, Katrin Steinhauer, Martin Exner, Nico T. Mutters

**Affiliations:** 1 Association for Applied Hygiene (VAH), Bonn, Germany; 2 Institute for Hygiene and Public Health. University Hospital Bonn, Bonn, Germany; 3 HygCen Germany GmbH, Schwerin, Germany; 4 Labor Prof. Dr. G. Enders MVZ GbR, Stuttgart, Germany; 5 Dr. Brill + Partner GmbH, Hamburg, Germany; 6 Eurofins Biolab Srl, Vimodrone, Italy; 7 Laboklin Labor für Klinische Diagnostik GmbH & Co. KG, Bad Kissingen, Germany; 8 Laboratoires Anios – an Ecolab company, Sainghin-en-Mélantois, France; 9 Expert Committee on Virus Disinfection of the German Association for the Control of Viral Diseases (DVV) e. V. and the Society for Virology (GfV) e.V., Heidelberg, Germany; 10 University Medicine Greifswald, Institute for Hygiene and Environmental Medicine, Greifswald, Germany; 11 Bactologicum GmbH, Itzehoe, Germany

**Keywords:** disinfectant, ring trial, reference substance, virucidal activity testing, glutaraldehyde, peracetic acid

## Abstract

**Introduction:**

Reference tests are crucial for ensuring the comparability and quality assurance of test results in the field of chemical disinfectant testing. For testing virucidal activity, EN 14476:2019 specifies formaldehyde as reference substance. However, due to toxicological and technical concerns, formaldehyde needs to be replaced with new and more suitable reference substances.

In order to find the replacement, in 2023-24 an international ring trial was conducted by the European Standardization Committee TC 216 WG 1 (chemical disinfectants and antiseptics, working group human medicine) with 17 participating laboratories. The goal was to evaluate the suitability of these substances for optimization of existing standards.

**Materials and methods:**

The study assessed the stability of the test viruses used in the disinfection test – specifically, Modified vaccinia virus Ankara, Adenovirus type 5, Murine Norovirus S99, Minute Virus of Mice, Poliovirus type 1 strain LSc-2ab and Bovine Enterovirus type 1 – to two newly selected reference substances: glutaraldehyde and peracetic acid.

The study tested reproducibility and repeatability based on a predefined test protocol.

**Results:**

The results of the two tested reference substances – glutaraldehyde and peracetic acid – provide new reference substance ranges for relevant test viruses. The findings are incorporated into the revision of the European standards, ensuring the quality assurance of efficacy testing in virucidal testing.

## Zusammenfassung

**Einleitung:**

Referenztests sind entscheidend für die Vergleichbarkeit und Qualitätssicherung von Prüfergebnissen im Bereich der chemischen Desinfektionsmittelprüfung. Für die Prüfung der viruziden Aktivität ist in der EN 14476:2019 Formaldehyd als Referenzsubstanz festgelegt. Aufgrund von toxikologischen und technischen Bedenken muss Formaldehyd jedoch durch neue und besser geeignete Referenzsubstanzen ersetzt werden.

Um einen Ersatz zu finden, wurde 2023-24 ein internationaler Ringversuch vom Europäischen Normungsausschusses TC 216 WG 1 (Chemische Desinfektionsmittel und Antiseptika, Arbeitsgruppe Humanmedizin) mit 17 teilnehmenden Laboren durchgeführt. Ziel war es, die Eignung dieser Stoffe für die Optimierung bestehender Normen zu bewerten.

**Material und Methode:**

Die Studie untersucht die Empfindlichkeit der im Desinfektionsmitteltests verwendeten Testviren – insbesondere Modifiziertes vaccinia virus Ankara, Adenovirus Typ 5, Murines Norovirus S99, Minute Virus of Mice, Poliovirus Typ 1 Stamm LSc-2ab und Bovines Enterovirus Typ 1 – gegenüber zwei neu ausgewählten Referenzsubstanzen: Glutaraldehyde und Peressigsäure. Im Rahmen der Studie wurden die Reproduzierbarkeit und Wiederholbarkeit auf der Grundlage eines vordefinierten Testprotokolls geprüft.

**Ergebnisse:**

Die Ergebnisse der beiden getesteten Referenzsubstanzen – Glutaraldehyde und Peressigsäure – lieferten neue Referenzbereiche für relevante Testviren. Diese Ergebnisse fließen in die Überarbeitung der europäischen Normen ein und gewährleisten die Qualitätssicherung von Wirksamkeitstests bei viruziden Prüfungen.

## Introduction

In Europe, chemical disinfectants must prove their activity in the tiered approach [1] using relevant surrogate test organisms that cover both enveloped and non-enveloped viruses. These tests comprise a quantitative suspension test (phase 2/step 1) and a test simulating practical conditions (phase 2/step 2). This approach ensures greater reliability of disinfectant activity against the target organisms. 

A unique feature of virucidal activity testing is the fact that it includes two combined biological systems – cells and viruses – each introducing multiple influencing factors. The standardization of these tests is crucial to ensure consistency [2]. Variations in the activity range, whether due to different virus sources, passages, or cell lines, highlight the need for reference tests that use a defined reference substance alongside the disinfectant product. This serves as an internal control for the test and the virus stability [1]. Reference test with formaldehyde is conducted with specific concentrations and contact times, which are intended to reduce the number of test viruses within a predefined range with phosphate buffered salt solution (PBS) as the interfering substance. Such tests ensure that a virus with defined stability is consistently used across variant tests, making results from a laboratory and between laboratories comparable.

Chemical reference substances are essential in efficacy testing, serving as standardized benchmarks to ensure test quality. Their role is particularly critical for maintaining the consistency of results and enabling objective comparisons of the activity of different disinfectants. The current European standard for virucidal activity testing (EN 14476:2019 [3]) specifies the use of a quantitative suspension test with an aqueous formaldehyde solution (0.7% w/v). 

Formaldehyde has historically been a favored chemical disinfectant due to its broad activity spectrum, stability, and material compatibility. The dramatic case of the Cutter incident in 1955, which showed that the methods used at the time to inactivate the poliovirus with formaldehyde were not always reliable, led to formaldehyde being adopted as a reference by many virologists in the 1970s, [4]. Many concerns about formaldehyde arose in the 1990s. In particular, the World Health Organization’s 2004 toxicological assessment, the setting of indoor exposure limits by the German Federal Institute for Risk Assessment [5], and its classification as a carcinogen have led to increasing scrutiny over the use of formaldehyde in (public) health care [6], [7]. Furthermore, its slow kinetics and the long contact times required for its disinfection efficacy make it unsuitable for everyday use, reinforcing the need for its gradual replacement [8]. 

The present ring trial aimed to confirm new potential reference substances to replace formaldehyde. First, suitable biocides must be selected and assessed for their potential as new reference substances in chemical disinfection testing. Specific requirements were established for these substances to ensure their suitability. Requirements for the implementation of reference substances:

- Relevance for products and test design
- Long-term availability for laboratories
- Stability (storage and quality) of the reference substance
- Possibility of analytical proof
- Level of the inactivation kinetic (steepness of inactivation kinetic according concentration steps)
- Acceptable variance of lg-reduction
- Simple neutralization (not suitable for viruses, only applicable for bacteria, yeasts and fungi)
- Safety for the user (laboratory personnel).

Both substances, glutaraldehyde and peracetic acid, fulfill the majority of the criteria and were selected as suitable substances for the CEN TC216 ring trial in 2023–24 [9]. 

Glutaraldehyde (GDA) is a well-established chemically active substance with extensive research supporting its biocidal properties [9]. Its chemical and physical parameters are thoroughly documented in the European Standards such as EN 16777:2018 [10], EN 17111:2018 [11] and DVV guideline 2012 [12]. For the CEN TC216 ring trial in 2023–24, data from an accredited French laboratory served as a basis for the test design to evaluate the replacement of formaldehyde with GDA in the standard. 

Additionally, peracetic acid (PAA) has been considered as an alternative reference substance. PAA has earned broad acceptance over decades due to its wide antimicrobial spectrum [9]; it is included in the sporicidal standards. The concentrations to be tested for virucidal activity were defined by the ring trial, drawing from a publication by Steinmann et al., which compared the efficacy of PAA against poliovirus and parvovirus [13]. 

The goal of the ring trial was not only to identify suitable reference substances but also to establish the lg-reduction range values for these alternatives. The introduction of new reference substances enables laboratories to use test organisms of similar stability. The results of the reference tests within the specified range are the basis for comparing the test results of products.

## Materials and methods

### Selection of participating laboratories 

In total, 17 international laboratories (Table 1 (Tab. 1)) participated in the ring trial for reference substances by order of CEN TC 216 (European Committee for Standardization – “Chemical disinfectants and antiseptics”), organized by the Association for Applied Hygiene (VAH) as the proficiency testing provider. Sixteen of the 17 laboratories were accredited. In total, a combination of the ring trial data and additional tests for specific viruses and concentration:time ratios carried out over a 1-year period with a high workload were analyzed and resulted in new ranges for virucidal reference substances. 

### Reference substances 

Two different possible new reference substances were chosen and tested in the CEN TC216 ring trial 2023–24. 

Sigma-Aldrich Glutaraldehyde (GDA) 50 wt.% water solution (Ref 340855, CAS number 111-30-8, DMT 2023-005) and peracetic acid (PAA) Lerasept Special 5% from STOCKMEIER CHEMIE (Ref 1000638701000, UFI: 2T5Y-71WN-Q00P-39SE) were provided from one batch to all participating laboratories by the VAH with the exact opening and filling date. The concentration of the reference solutions prepared in water (water for injection [WFI]) according to EN 14476:2019 [3] was 1.25 times the desired test concentration because of dilution during the test (Table 2 (Tab. 2)). 

The test temperature of the GDA solution was adjusted to 20°C before any use. A stock solution of 1% (v/v) dilution of glutaraldehyde (50%) was freshly prepared before every test using WFI. The PAA solution was equilibrated to the test temperature of 10°C before any use. A stock solution of 0.1% (v/v) dilution of peracetic acid (5%) was freshly prepared before every test using WFI. With each prepared stock solution, the reference substance dilutions were prepared exactly according to Table 2 (Tab. 2). 

### Test viruses and cell culture 

All relevant viruses of various European standards were tested, and the results will be relevant across all working groups of the CEN TC216. The quantitative suspension test was performed using the following six test viruses according to the test design:

1. Modified vaccinia virus Ankara (MVA), ATCC-VR-1508 (one laboratory used the vaccinia virus Elstree strain)
2. Adenovirus 5 (AV5); strain adenoid 75, ATCC-VR-5
3. Murine norovirus (MNV), strain S99 Berlin provided by the Friedrich Loeffler Institute
4. Murine parvovirus, minute virus of mice (MVM), strain Crawford, ATCC VR-1346
5. Poliovirus type 1 Sabin vaccine strain [LS-c, 2ab strain](PV-1) provided by the Friedrich Loeffler Institute
6. Bovine enterovirus type 1 (ECBO for enteric cytopathic bovine orphan *virus)* VR-248.

Table 3 (Tab. 3) documents the cell culture used for each test virus is documented for the participating laboratories. For the evaluation of adenovirus (AV5), only the results from laboratories which used HeLa-cells or derivate were used for the definition of the lg-reduction range of the reference substance (all data for AV5 with different cell lines can be found in Attachment 1). The media, which were used for the individual cell cultures, were selected independently by the laboratories. 

### Test procedure 

The virucidal efficacy of several concentrations of GDA and PAA were tested in the quantitative suspension test according to EN 14476:2019 [3] against the test viruses, but only the relevant data for defining a lg-reduction range are presented (Table 4 (Tab. 4)). These tests were performed with phosphate buffered salt solution (PBS according to EN 14476:2019 5.2.2.3 [3]) as interfering substance at 20°C with GDA and 10°C with PAA. 

All substances were adjusted to the respective test temperature (here 20°C±1°C or 10°C±1°C) before tests started. 

Interfering substance (1 part PBS) and the virus test suspension (1 part) were mixed and the corresponding reference substance (8 parts GDA/PAA) was added to the mixture. After the respective contact time at the respective test temperature, infectivity was determined using the endpoint-titration method. Immediately after the chosen contact time, a series of ten-fold dilutions was prepared down to 10^–8^. Afterwards, each dilution was transferred (0.1 ml) into eight wells of a microtitre plate containing a confluent (>90%) cell monolayer or suspension cells. After incubation, the virus titre was measured as the tissue culture infection dose 50 per milliliter (lg TCID_50_/ml). 

In addition to the actual test procedure, the following controls were carried out. For the cell control, culture medium (0.1 ml) was added to the last row of each microtiter plate. The virus control had to be determined under test conditions at the beginning of the contact time (VC T0) and after each contact time chosen (VC Tx). The cytotoxicity [lg CD_50]_ of GDA or PAA was examined for all concentrations tested. Cell culture results were documented as “0” for absence of a cytopathic effect (CPE) and as “1” (approximately 25% of cells with CPE) to “4” (all cells with CPE) for the endpoint titration. In case no virus replication was observed in the highest concentration, this value was preceded by “≤”. 

### Statistics 

Prior to the evaluation according to DIN EN ISO 13528:2020-09 [14], all results were reviewed for plausibility by the proficiency testing provider VAH. The values calculated by the test provider were based on the submitted raw data. 

Virus titers were determined using the method of Spearman [15] and Kaerber [16], expressed as lg TCID_50_/ml. The efficacy of the reference substance was assessed by calculating the lg-reduction (lg R) with a 95% confidence interval. The lg-reduction represents the difference in infectivity titer (lg TCID_50_/ml) between the virus control obtained without exposure to the disinfectant and the titer after exposure to the reference substance according to EN 14476:2019 [3].

A robust statistical method, based on DIN EN ISO 13528:2020-09 [14], was applied for the performance evaluation. All statistical analyses were performed using PROLab standard version 2021.7.22.0 software. Laboratory performance was assessed by applying z-scores to identify variations between laboratories and support method standardization.

**Figure 1:**
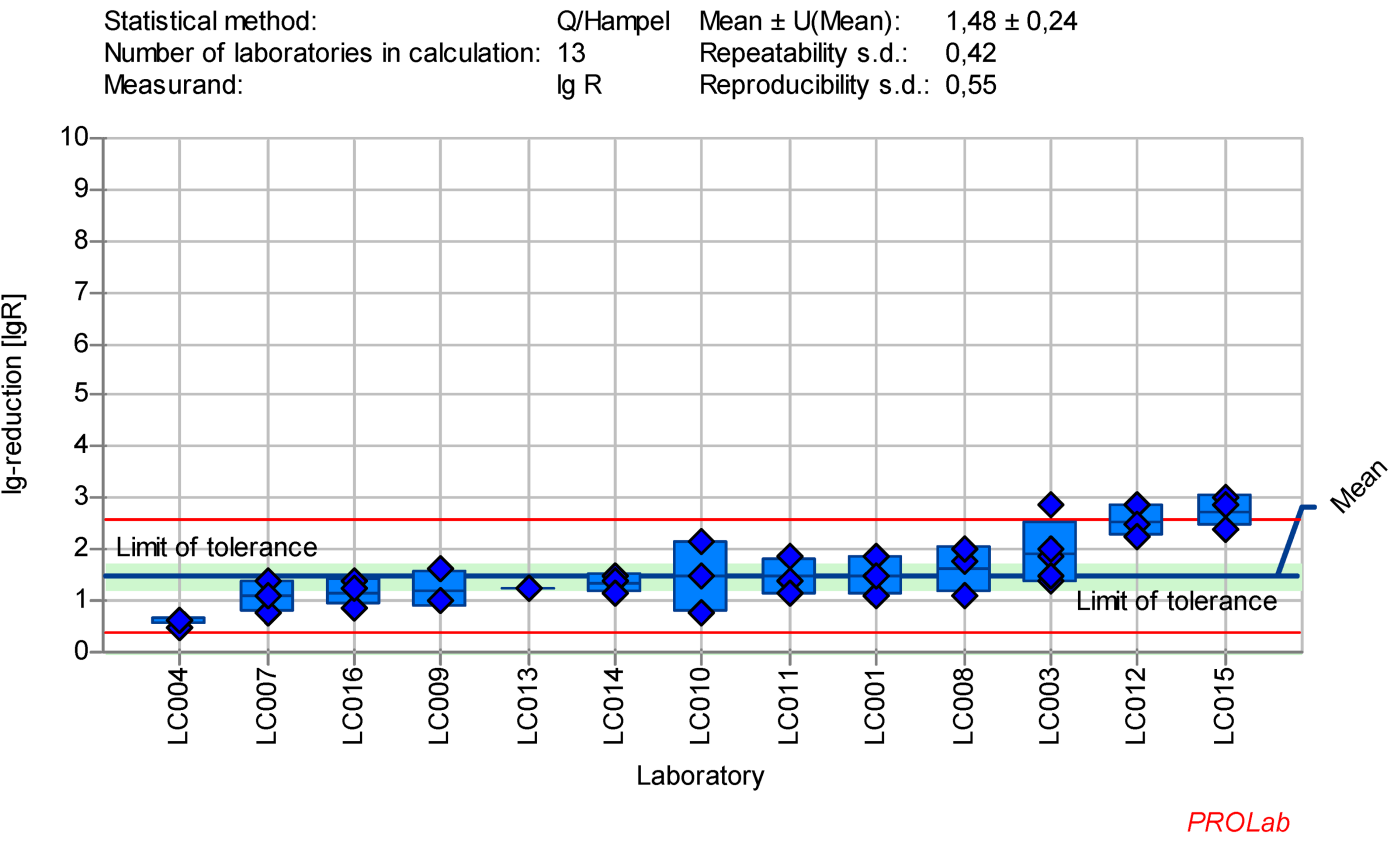
Mean and standard deviation of the lg-reduction for MVA with glutaraldehyde 50 ppm:5 min sorted by laboratory mean values (mean lg R for defining the range without LC015 is 1.40±0.23)

**Figure 2:**
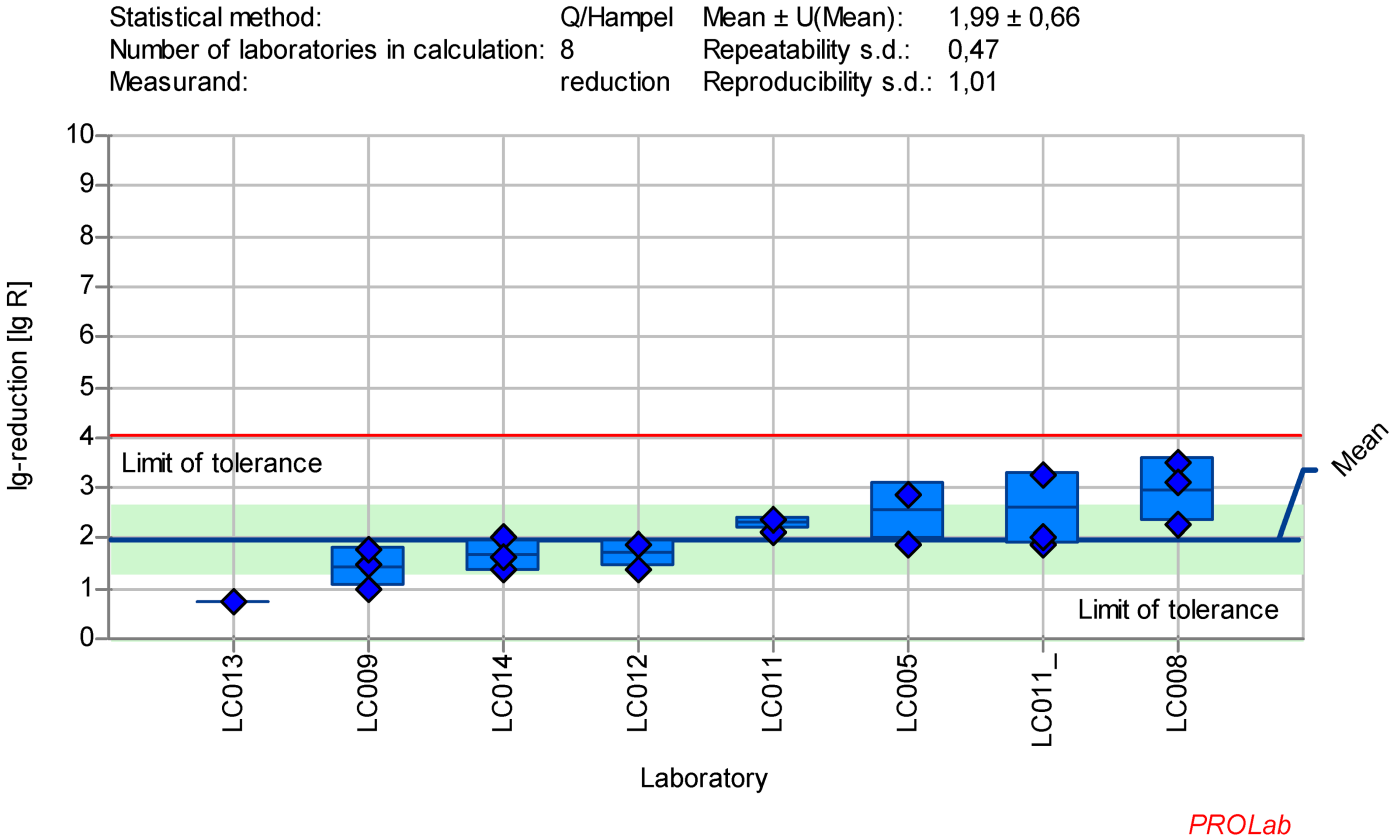
Mean and standard deviation of the lg-reduction for AV5 (cultivation only with HeLa cells or derivate) with glutaraldehyde 50 ppm:30 min sorted by laboratory mean values (LC011_ used Hep-cells; mean lg R for defining the range is 1.99±0.66)

**Figure 3:**
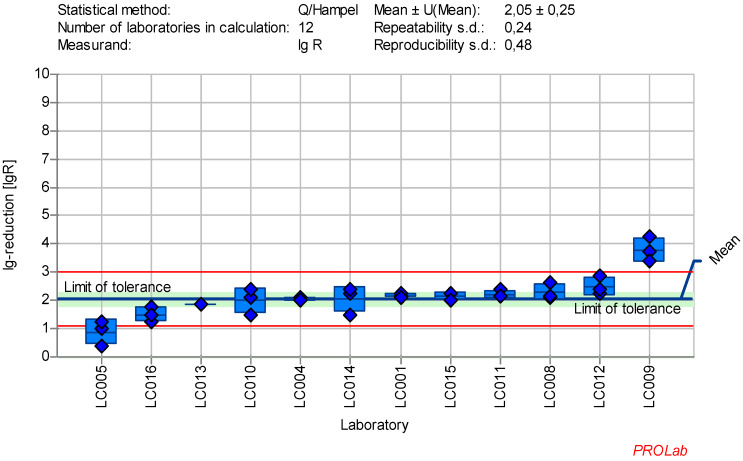
Mean and standard deviation of the lg-reduction for MNV with glutaraldehyde 100 ppm:30 min sorted by laboratory mean values (mean lg R for defining the range without LC005 and LC009 is 2.09±0.15)

**Figure 4:**
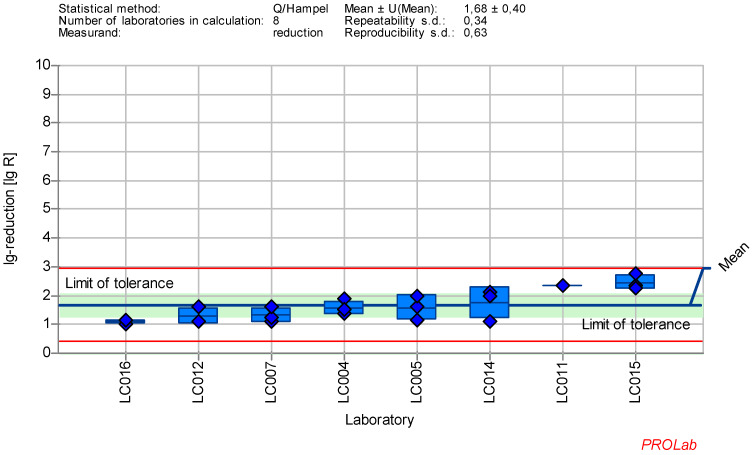
Mean and standard deviation of the lg-reduction for MVM with glutaraldehyde 500 ppm:30 min sorted by laboratory mean values (mean lg R for defining the range is 1.68±0.40)

**Figure 5:**
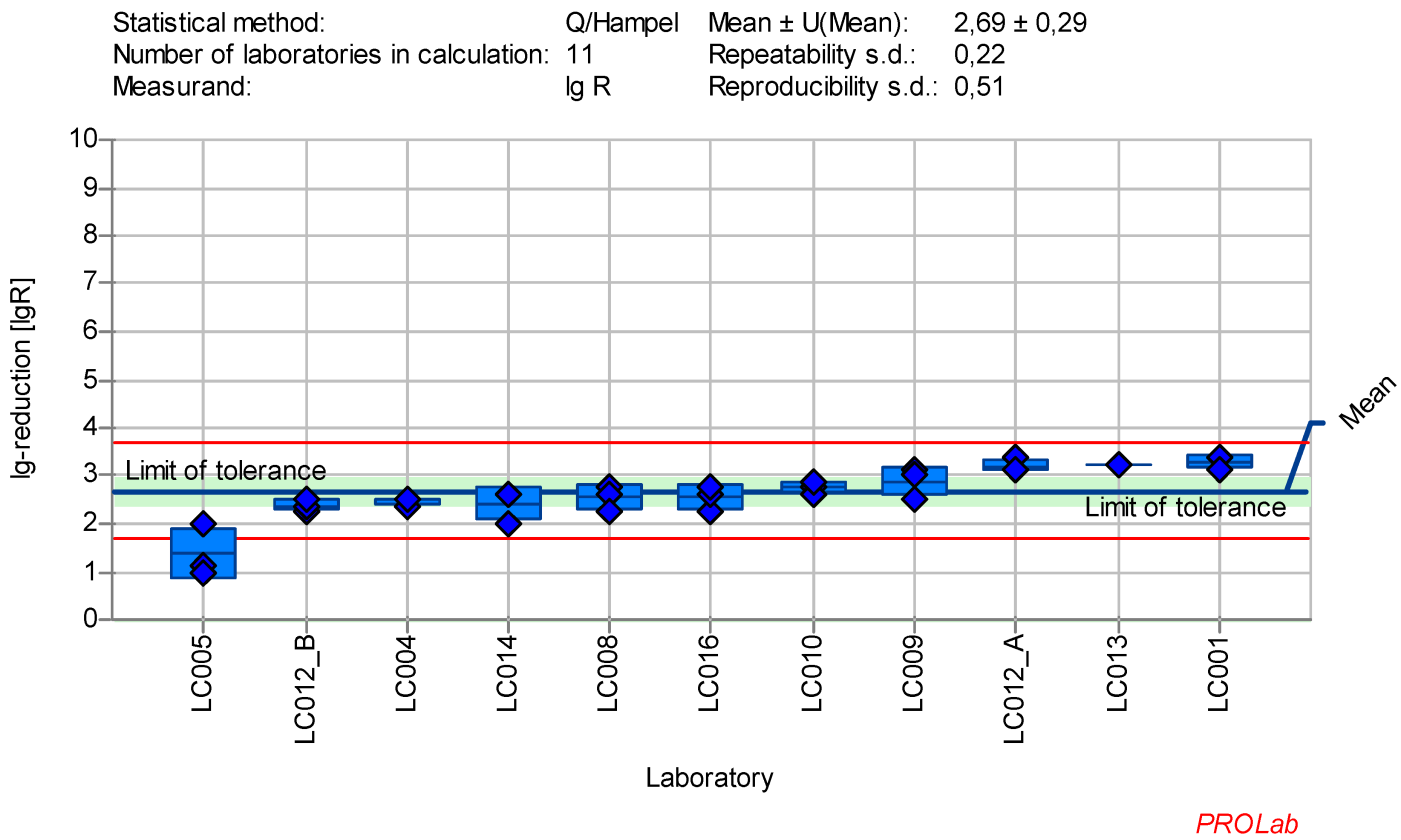
Mean and standard deviation of the lg-reduction for poliovirus with glutaraldehyde 500 ppm:30 min sorted by laboratory mean values (mean lg R for defining the range without LC005 is 2.77±0.29)

**Figure 6:**
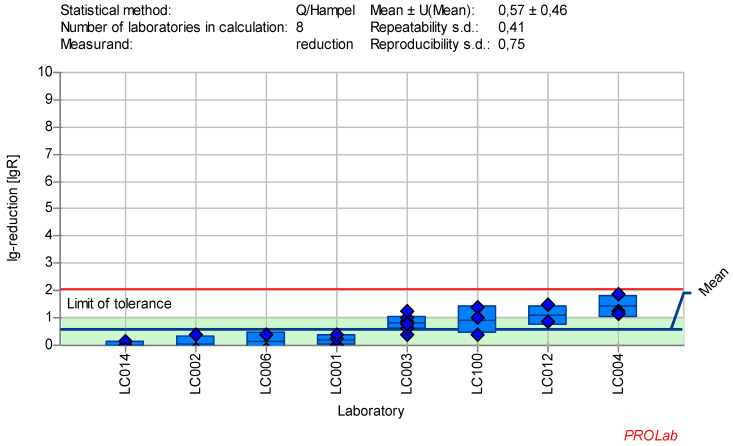
Mean and standard deviation of the lg-reduction for ECBO with peracetic acid 25 ppm:30 min sorted by laboratory values (mean lg R for defining the range is 0.57±0.46)

**Figure 7:**
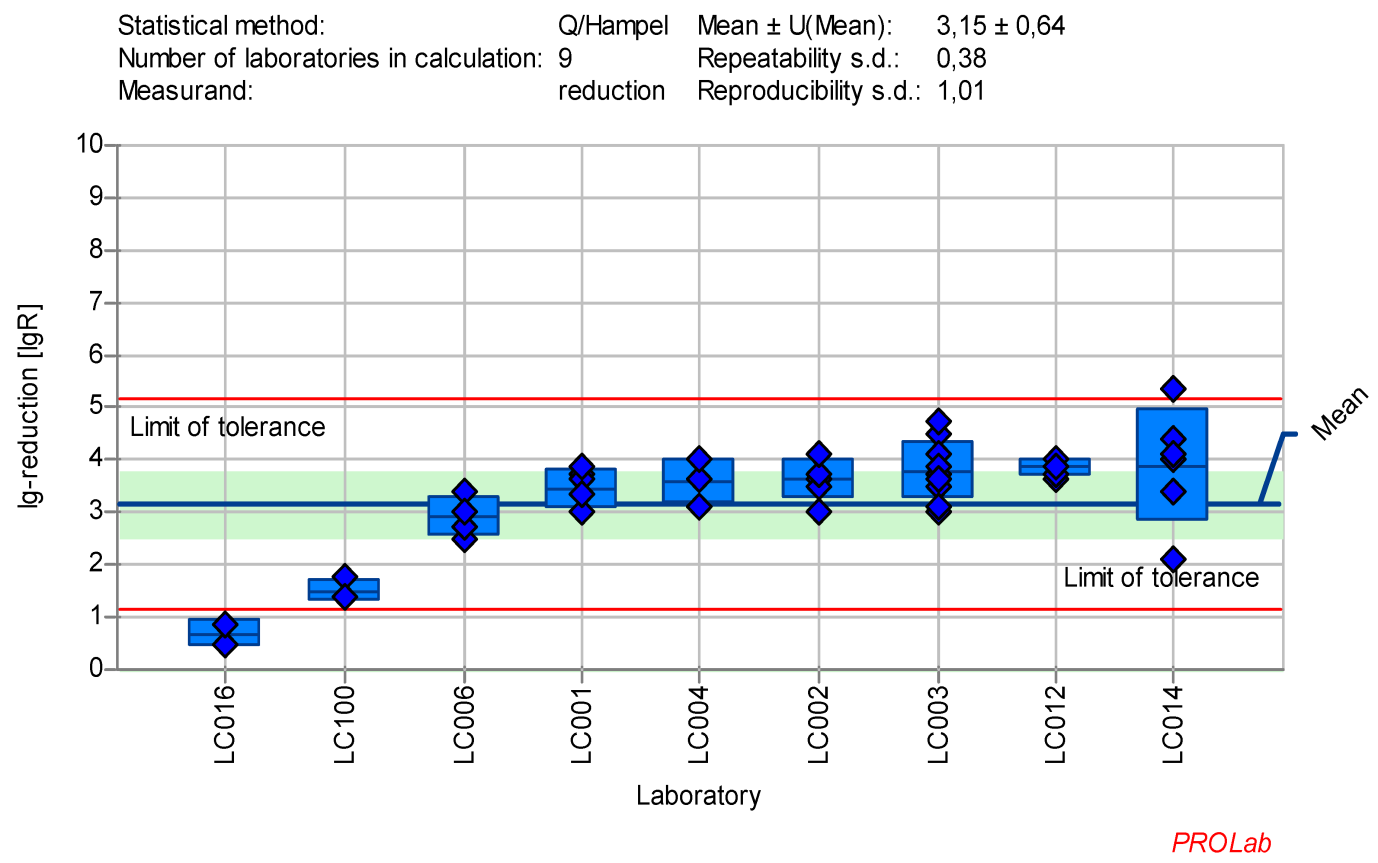
Mean and standard deviation of the lg-reduction for ECBO with peracetic acid 100 ppm:30 min sorted by laboratory values (mean lg R for defining the range without LC016 is 3.43±0.46)

The individual lg-reduction results for each laboratory and reference substance are displayed in Figure 1 (Fig. 1), Figure2 (Fig. 2), Figure 3 (Fig. 3), Figure 4 (Fig. 4), Figure 5 (Fig. 5), Figure 6 (Fig. 6), Figure 7 (Fig. 7). It should be noted that not all data were included in the determination of the lg-reduction range. Larger boxes indicate higher variability in lg-reduction, with the horizontal line representing the laboratory mean value. Small crossed-out circles represent individual measurements. Tolerance limits are set at ±2 times the reproducibility standard deviation, and if the lower limit falls below zero, the red line is omitted, with zero considered the lower limit. 

The following statistical requirements were applied to the CEN TC216 ring trial 2023–24 results before defining lg-reduction ranges for the reference substances:

- A minimum of eight participating laboratories is required for statistically reliable results (DIN ISO 13528:2020-09 [14]).
- The repeatability value (internal lab deviation value) should not exceed 0.5.
- The key statistical value of defining a reference substance range is reproducibility, reflecting deviation across all laboratories [17]. While chemical analysis typically requires reproducibility ≤0.5 (ISO TS 22117:2019-08 [18]), a higher value is justifiable in virucidal testing due to the two distinct microbiological systems involved (test virus and cell culture). Based on experience of the participating laboratories, a reproducibility value of ≤1.0 is acceptable for a robust dataset.

These criteria establish the basic requirements for using ring trial data to define lg-reduction ranges for each test virus strain and parameter in reference testing standards. 

## Results

17 laboratories provided data for the evaluation of new, potential reference substances in virucidal activity testing. Most laboratories met the required acceptance criteria for evaluation of the submitted data. The titer of the test suspension had a lg TCID_50_ value of 10^8^ per ml or at least a concentration sufficient to allow the determination of a 4 lg-reduction of the virus titer. All raw data were evaluated, but only the results which yielded a definition of the lg-reduction range for the potential, new reference substances, are shown in this publication. 

According to EN 14476:2019 [3] standard, the cytotoxicity of the product should not affect cell morphology, growth or stability of the test viruses in the dilutions necessary to demonstrate a 4 lg reduction of the virus. No statistically significant performance differences were found between laboratories that reported cytotoxicity and those that did not. However, as the concentration increased, most laboratories observed higher cytotoxicity (Table 5 (Tab. 5)).

### Range of virus test suspension, virus control T0 and Tx according to EN 14476:2019 

In the following, the mean virus titer of virus test suspension, the virus control at the beginning of the contact time (VC T0) and after each contact time (VC Tx) of all test viruses are presented in Table 6 (Tab. 6), Table 7 (Tab. 7), and Table 8 (Tab. 8). All laboratories achieved an adequate virus test suspension of at least 10^8^ per ml or a concentration sufficient to allow the determination of a 4-lg-reduction in the virus titer (Table 6 (Tab. 6)). The requirements for repeatability and reproducibility values were met for the virus test suspension, with repeatability ≤0.5 and reproducibility ≤1.0 (Table 6 (Tab. 6)). The virus control at T0 should recover approximately 1-lg level fewer virus than the virus test suspension, due to dilution. Fluctuations must be taken into account, but this criterion was met for almost all viruses (Table 7 (Tab. 7)). As a contact time of 30 min was selected for all viruses, except MVA, the virus control data is stable across viruses. When comparing the virus controls at the beginning and end of the contact time, hardly any differences were observed (Table 8 (Tab. 8)). 

### Statistical parameters of the lg-reduction 

In the following, the statistical parameters for all viruses for the quantitative suspension test with the reference substances are given. Here, only data which resulted in the new ranges for reference substances are shown.

Table 9 (Tab. 9) includes the mean value of the lg-reduction and the robust reproducibility and repeatability value for each virus under the specific condition. In almost all cases, the repeatability value of ≤0.5 and the reproducibility value of ≤1.0 for the tested reference substance indicates high repeatability and reproducibility. For the test virus AV5, the reproducibility is slightly over 1, due to general problems with AV5 as a test virus. Additional information can be found in. 

### Reduction of modified vaccinia virus Ankara (MVA) with glutaraldehyde 

The dataset of 13 laboratories in the ring trial for MVA with GDA at a concentration:time ratio of 50 ppm:50 min produced the best reproducible results in terms of the established criteria for reference substances (Figure 1 (Fig. 1)). The following exceptions must be taken into account:

- The viruses were tested according to the protocol, with the exception of one laboratory which used vaccinia virus Elstree instead of MVA. While the choice of the virus is optional according to the standard, vaccinia virus Elstree is known to be more sensitive [19], so the results from this laboratory were excluded from the statistical analysis in this publication.
- Only one laboratory (LC015) triggered a warning signal in the z-score analysis for the lg-reduction of MVA at the 50 ppm:5 min concentration:time ratio. As a result, this outliner was excluded from the definition of the lg-reduction range for MVA. All other laboratories showed consistent results and were included in the definition of a lg-reduction range for GDA with MVA. The mean lg-reduction of MVA, excluding LC015, is 1.40±0.23 with repeatability value of 0.40 and reproducibility value of 0.52. These values were used to define the lg-reduction range for MVA with GDA.

### Reduction of adenovirus (AV5) with glutaraldehyde 

The dataset in the ring trial for AV5 with GDA exhibited significant variability, preventing the definition of a suitable range for the reference substance. As a result, additional tests were conducted, and concentration:time ratios were adjusted. 

It was observed that different cell lines were used for AV5 testing (Table 4 (Tab. 4)), which could account for some of the large deviations in the results (see Attachment 1). Notably, laboratories using HeLa cells (or Hep-2, a derivative of HeLa cells) showed smaller deviations, which was confirmed by the absence of abnormalities in the z-score analysis. When analyzing the results from eight laboratories using HeLa cells with 50 ppm GDA for 30 minutes, the data met the criteria for reference substances (Figure 2 (Fig. 2)).

### Reduction of murine Norovirus (MNV) with glutaraldehyde 

The dataset of 12 laboratories in the ring trial for MNV with GDA at a concentration:time ratio of 100 ppm:30 min yielded the best reproducible results in terms of the established criteria for reference substances (Figure 3 (Fig. 3)). The following exceptions must be considered:

- The z-scores for the lg-reductions of MNV showed one warning signal for laboratory LC005 and one action signal for laboratory LC009. As a result, these two outliners were excluded from the definition of the lg-reduction range. All other laboratory results were included in the definition of the lg-reduction range.
- The mean value of the lg-reduction of MNV, excluding LC005 and LC009, is 2.09±0.15 with a repeatability value of 0.23 and a reproducibility value of 0.31. These values were used to define the new lg-reduction range for MNV with GDA.

### Reduction of murine Parvovirus (MVM) with glutaraldehyde 

The dataset of 8 laboratories in the ring trial for MVM with GDA at a concentration:time ratio 500 ppm:30 min showed the best reproducible results regarding the established criteria for reference substances. As no anomalies were found in the z-score analysis, all data of the laboratories were included in the definition of the lg-reduction range of MVM with GDA (Figure 4 (Fig. 4)). 

### Reduction of PV-1 virus Type 1 with glutaraldehyde 

The dataset of 11 laboratories in the ring trial for poliovirus with GDA at a concentration-time ratio 500 ppm:30 min produced the best results according to the established criteria for reference substances (Figure 5 (Fig. 5)). The following exceptions must be taken into account:

- A warning signal for the lg-reduction of poliovirus was generated by one laboratory (LC005) and therefore had to be excluded for the definition of the lg-reduction range for poliovirus.
- The mean lg-reduction for poliovirus, excluding LC005, is 2.77±0.29 with a repeatability value of 0.21 and a reproducibility value of 0.49. These values were used to define the new lg-reduction range for poliovirus with GDA.

### Reduction of bovine Enterovirus type 1 (ECBO) with peracetic acid 

For ECBO, various concentration-time relations of glutaraldehyde were tested, but did not show increasing kinetics with increasing concentration (<1 lg reduction). The criteria for reference substances and their testing were not met; therefore the data turned out to be unsuitable for reference tests.

Therefore, an alternative reference substance was needed. Peracetic acid (PAA) was selected, as it is already established as a reference substance and is effective against ECBO, an ability that few other active ingredients possess. Additionally, PAA is not only widely used in the human medical area, but also in the veterinary sector. Different concentrations of PAA were tested by each laboratory, with concentrations chosen individually to produce data near the borderline range. It was observed that PAA exhibits a very rapid kinetic profile, making it challenging to define a single concentration range in which the virus is consistently detectable.

To address this, a revised test design was developed to evaluate two concentrations: one below and one above a specified value. This approach is similar to the one used for PAA with bacterial spores as outlined in EN 17126:2019-02 [20]. The task force decided to define two concentration ranges for PAA, based on the data obtained from 25 ppm PAA:30 min and 100 ppm PAA:30 min. 

All lg-reduction results for the 25 ppm PAA:30 min concentration:time ratio were included in the definition of the lower value (Figure 6 (Fig. 6)). However, as the z-score analysis revealed abnormalities for one laboratory (LC016), this laboratory’s data was excluded from the definition of the lg-reduction range for the higher concentration of 100 ppm PAA. The revised mean lg-reduction for ECBO at 100 ppm PAA:30 min, excluding LC016, is 3.43±0.46, with a repeatability value of 0.38 and a reproducibility of 0.75. These values were used to define the new lg-reduction range for ECBO with PAA (Figure 7 (Fig. 7)).

## Discussion

Since 1989, the Technical Committee 216 (TC 216) “Chemical disinfectants and antiseptics” of the European Committee for Standardization (CEN) has been developing methods to test the activity of disinfectants in Europe [21]. These methods are continuously reviewed and refined to improve product quality and ensure user safety [2]. 

Historically, formaldehyde was the sole reference substance for virucidal testing. However, due to its high cytotoxicity, carcinogenic classification, and associated toxicological concerns, formaldehyde is no longer approved for use in virucidal activity testing in many countries [22]. Due to the lack of specifications or regulations on the part of the countries in which formaldehyde is not approved, those laboratories must create alternatives based on their own data. If replacement is not possible, formaldehyde exposure must be reduced by air filtration or individual protection against exposure. Given the growing demand for alternatives, particularly in countries with stringent regulations, data from France was used to design a new test protocol for the ring trial. 

This is the first independent, large-scale international ring trial testing virucidal reference substances across 17 laboratories, establishing a statistically robust foundation for virucidal activity testing across relevant test viruses. The data collected met the quality and statistical criteria required for reference substances. The importance of a well-organized, broad-based ring trial in improving standards cannot be overstated. The active participation of laboratories and the collaborative evaluation among experts have proven invaluable in generating new data and insights, ultimately enhancing the quality of the testing process. The ring trial met the criteria for reference substances and their testing. 

The new reference substances – glutaraldehyde (GDA) and peracetic acid (PAA) – were selected by the task force for reference substances of CEN TC 216 WG 1, as both met the requirements for reference substances. Both GDA and PAA are active ingredients in commonly used products, well-characterized, and effective against relevant pathogens, making them highly appropriate for disinfectant testing. GDA offers numerous advantages, including broad biocidal properties and excellent material compatibility, but is classified as a substance to be replaced in the future (BPR). Despite this, its cytotoxicity and toxicological risks to laboratory personnel are minimal and can be effectively managed with proper safety guidelines. The consistency and quality of both substances across batches (Sigma Aldrich and Lerasept spezial from STOCKMEIER CHEMIE) were carefully monitored to ensure reliable and reproducible results. This also included ensuring stable storage conditions, practical handling, and long-term availability. By utilizing well-defined and widely recognized reference tests, laboratories worldwide can compare their results, which helps ensure the consistency of test results, while also determining the stability of the test organisms. Improving the quality of test results increases the safety for users and patients. 

The data collected in the ring trial met the required quality criteria and statistical requirements for reference substances. The chosen concentrations of the reference substances did not affect the cell morphology or influence the evaluation process (Table 5 (Tab. 5)). Moreover, the virus test suspension, and virus control at the beginning of the contact time (VC T0) as well as after each contact time (VC Tx) according to EN 14476:2019 [3] were within the limit of tolerance. Figure 1 (Fig. 1), Figure2 (Fig. 2), Figure 3 (Fig. 3), Figure 4(Fig. 4) , Figure 5 (Fig. 5), Figure 6 (Fig. 6), and Figure 7 (Fig. 7) demonstrate that at least eight laboratories were within the tolerance limits for lg-reductions of each test virus, with only a few laboratories excluded from the definition of the ranges. As shown in Table 9 (Tab. 9), the repeatability value for almost all laboratories and reproducibility across laboratories for lg-reduction met the statistical requirements for reference substances (see Introduction) and the statistical requirements for reference tests (see reduction and statistics sections). The repeatability value was ≤0.5, and the reproducibility value was ≤1.0 for most test viruses, except for AV5 (with a reproducibility value of 1.01). The analysis of AV5 data was challenging due to significant variation both within and between laboratories (see Attachment 1). Adjustments to the concentration:time ratios, did not resolve the issue. Ultimately, one factor was eliminated as only results based on HeLa-cells (standard cell culture for EN 14476:2019 [3]) were included in the analysis. As HeLa cells were predominantly used in this study, the focus remained on these results. If future data suggests otherwise, the ranges may need to be revised or the cell culture specified. Nevertheless, the AV5 dataset remains statistically solid with at results from at least eight laboratories (Table 9 (Tab. 9)). The use of other cell lines is not excluded as long as they are comparable to HeLa cells and fulfill these requirements.

With the specific lg-reductions for each concentration:time ratio of the reference substances for each test virus, new lg-reduction ranges have been defined. If the statistical requirements for reference substances are met for a test virus under specified test conditions, the mean lg-reduction for each virus strain and contact time was taken, and the range was defined with ±1 lg from this mean value. Consequently, a 2-lg reduction range is defined for all test virus (except ECBO). As lg-reductions of 0 lg or 4 lg are not suitable as desired lg-reductions, the ranges should be between 1 lg and 3 lg. This allows reproduction and enables the stability of the viruses to be maintained at a constant level. 

For all tested viruses (except ECBO), the chosen concentration:time ratio met the criteria for defining new reference substance ranges. For ECBO, PAA was tested at two concentrations; thus, the definition of limits was adjusted to reflect two separate defined values: one for the upper limit and one for the lower limit (Table 10 (Tab. 10)).

The new reference substances and their corresponding lg-reduction ranges for virucidal activity testing were incorporated into the FprEN 14476:2024 (draft for formal vote) [23] as well as the FprEN 17914:2024 [24]. This success must be sustained, and reference substances should continue to be tested under practical conditions. New reference substances must also be gradually introduced to cover additional active substances. The development of new reference substances that meet both the general and statistical criteria is a significant advancement in quality control. Enhancing the reliability and predictive power of test results will ultimately improve public health and safety.

## Conclusions

The international CEN TC216 ring trial study conducted by European Standardization Committee TC 216 WG 1 and WG 5 (2023–24) successfully identified GDA and PAA as suitable alternatives to formaldehyde for virucidal testing. The establishment of new lg-reduction ranges for all relevant test viruses, based on reproducibility and repeatability tests, marks a significant advancement in disinfection testing. These new reference substances and their ranges are incorporated into the FprEN 14476:2024 [23] and FprEN 17914:2024 [24] and will soon become mandatory, maintaining the reliability and quality assurance of virucidal efficacy testing. This initiative lays the framework for providing reference substances and ranges into other areas of disinfectant testing, thereby contributing to improved public health and safety.

## Notes

### Competing interests

The authors declare that they have no competing interests.

### Funding 

This work was funded by the Association for Applied Hygiene (VAH), Germany. 

### Acknowledgments 

The authors are grateful for the input provided by all members of the Task Force of reference substances of the WG 1 “Human medicine” of the European Committee for Standardization (CEN) Technical Committee 216 for “Chemical disinfectants and antiseptics” under the leadership of Task Force leader Claudia Hildebrandt. 

Special thanks go to the laboratories which participated in the ring trial 2023-24 for their input in this important work.

### Authors ORCIDs:

- Roesch KM: https://orcid.org/0009-0000-3494-9191
- Hildebrandt C: https://orcid.org/0000-0001-7608-6670
- Eggers M: https://orcid.org/0000-0001-8485-9485
- Brill F: https://orcid.org/0000-0001-9681-8752
- Cavalleri M: https://orcid.org/0000-0001-5308-1201
- Gebel J: https://orcid.org/0000-0001-9328-3174
- Loeffert S: https://orcid.org/0000-0002-0698-8000
- Rausch M: https://orcid.org/0000-0003-1562-4337
- Schwebke I: https://orcid.org/0009-0007-6708-5845
- Steinhauer K: https://orcid.org/0000-0002-4218-6152
- Exner M: https://orcid.org/0000-0002-6383-7866
- Mutters NT: https://orcid.org/0000-0002-0156-9595

## Supplementary Material

Supplementary data

## Figures and Tables

**Table 1:**
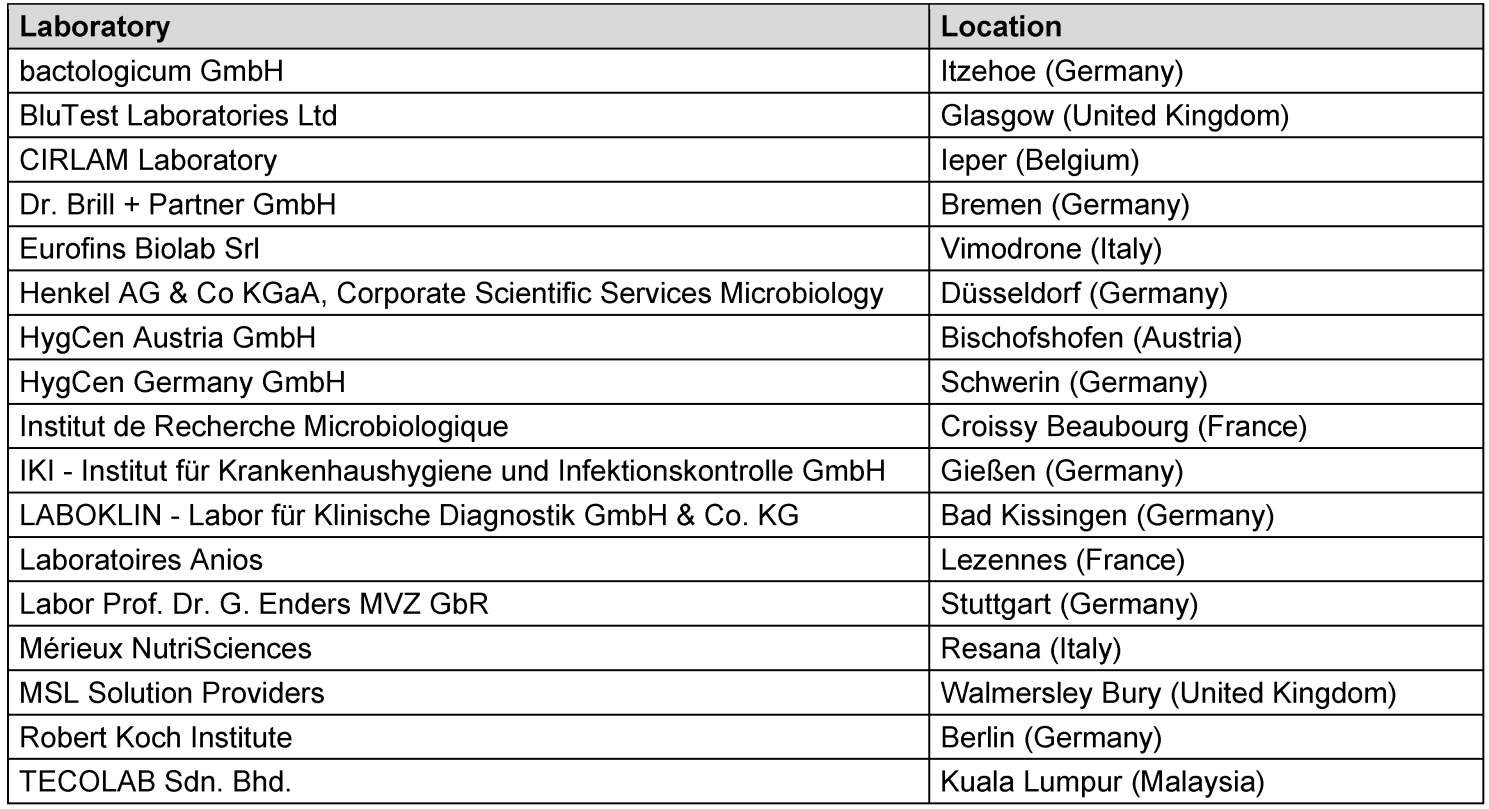
Participants of the CEN TC216 ring trial 2023–24 with reference substances

**Table 2:**
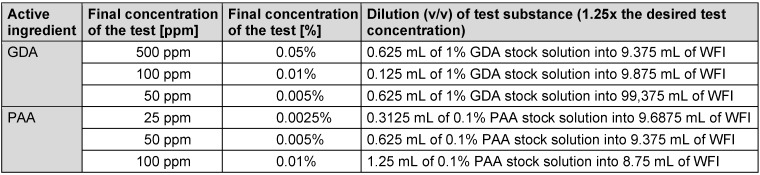
Guide for preparation of reference solutions (v/v) glutaraldehyde and peracetic acid

**Table 3:**
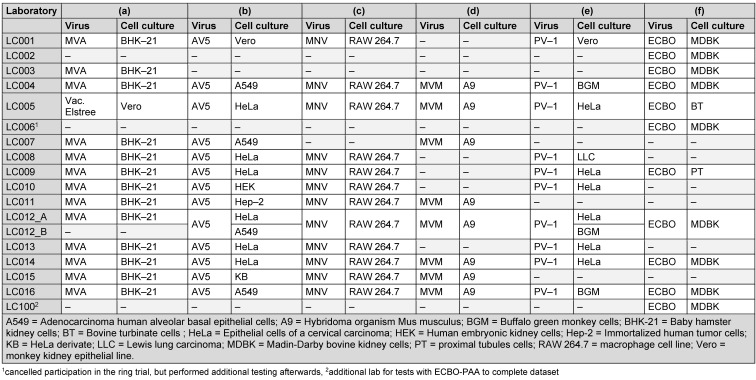
List of test viruses with the cell cultures used, sorted by laboratory [“–“= not done]

**Table 4:**
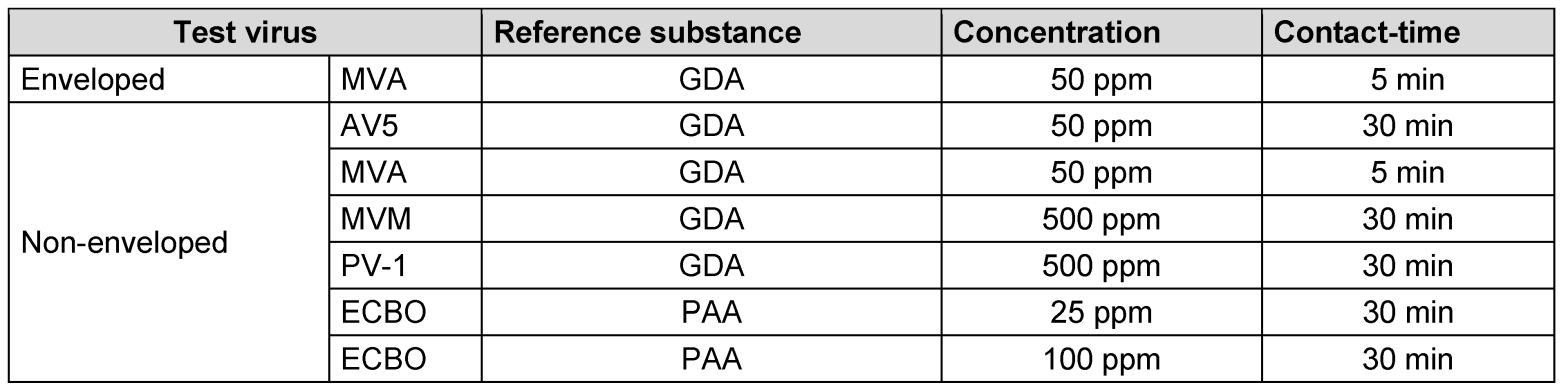
Final concentration and contact times for each reference substance and test viruses agreed by the task force for reference substances

**Table 5:**
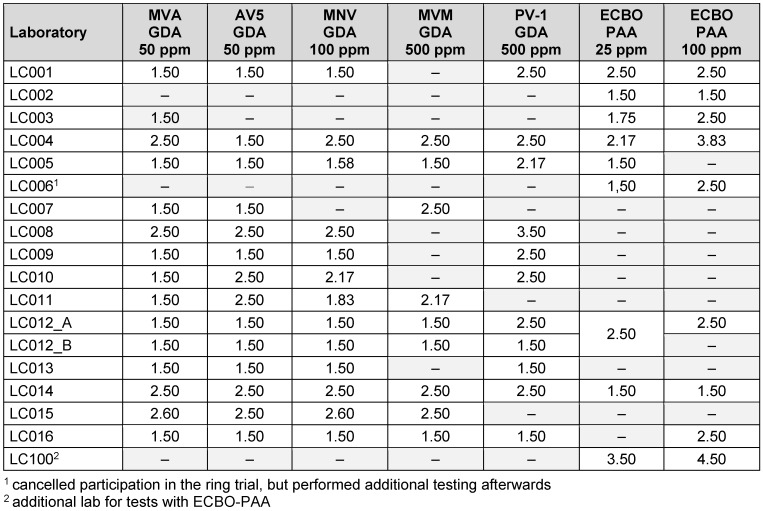
Mean cytotoxicity [lg CD_50_/ml] of glutaraldehyde and peracetic acid reported by laboratories (“–” = not done; _A/_B indicates different cell lines)

**Table 6:**
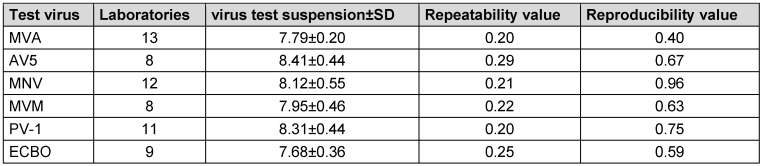
Mean value with the standard deviation (± SD) of virus titer of virus test suspension with the number of laboratories, repeatability and reproducibility values (lg TCID_50_/ml)

**Table 7:**
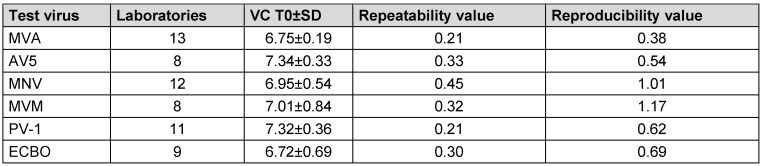
Mean value with the standard deviation (±SD) of virus control VC at the beginning of the contact time T0, with the number of laboratories, repeatability and reproducibility [lg TCID_50_/ml]

**Table 8:**
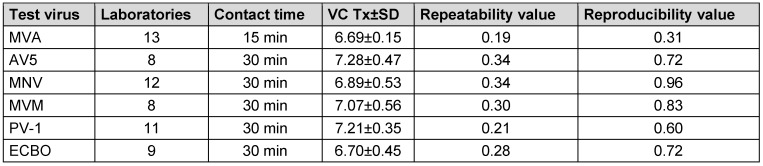
Mean value with the standard deviation (±SD) of virus control VC after each contact time Tx, with the number of laboratories, repeatability and reproducibility [lg TCID_50_/ml]

**Table 9:**
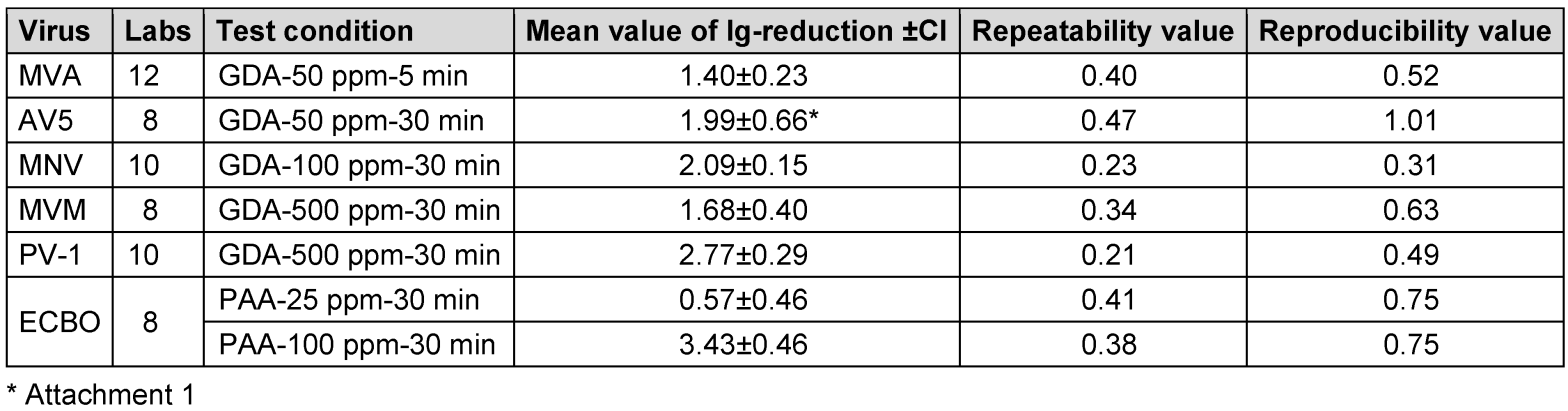
Repeatability and reproducibility values for the mean values of lg-reduction, which were used for defining the lg-reduction range, for all test viruses against the reference substance according to the protocol (CI=confidence interval).

**Table 10:**
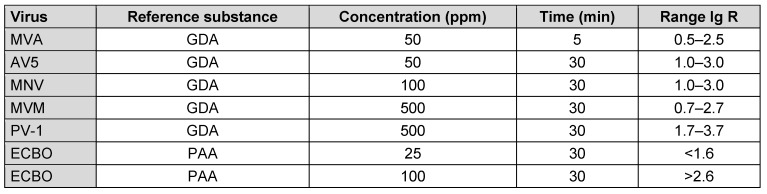
Final and agreed reference substances and lg-reduction ranges for virucidal activity testing
