# Supplementary material for: Enhancing the validity of virucidal activity testing of chemical disinfectants by establishing new reference substances according to EN 14476:2019
Source: GMS Hyg Infect Control. 2025 Sep 9;20:Doc50. doi: 10.3205/dgkh000579 (PMC12587039; doi:10.3205/dgkh000579)
Supplement: Supplementary data [file HIC-20-50-s-001.pdf]

Attachment 1

Supplementary data

Reduction of adenovirus (AV5) with glutaraldehyde

The dataset of 17 laboratories in the ring trial with additional tests for adenovirus with glutaraldehyde at the concentration:time ratio 50 ppm:30 min showed variable results. Different cell cultures were used (see Table 3 in main article).

The mean value of the lg-reduction of AV5 is 2.22 ± 0.75 with a repeatability value of 0.44 and a reproducibility of 1.59 is too high (≥ 1) for the definition of a new lg-reduction range for AV5 with GDA. The statistical requirement for the definition of the lg-reduction range for AV5 for the reference substance GDA is not met.

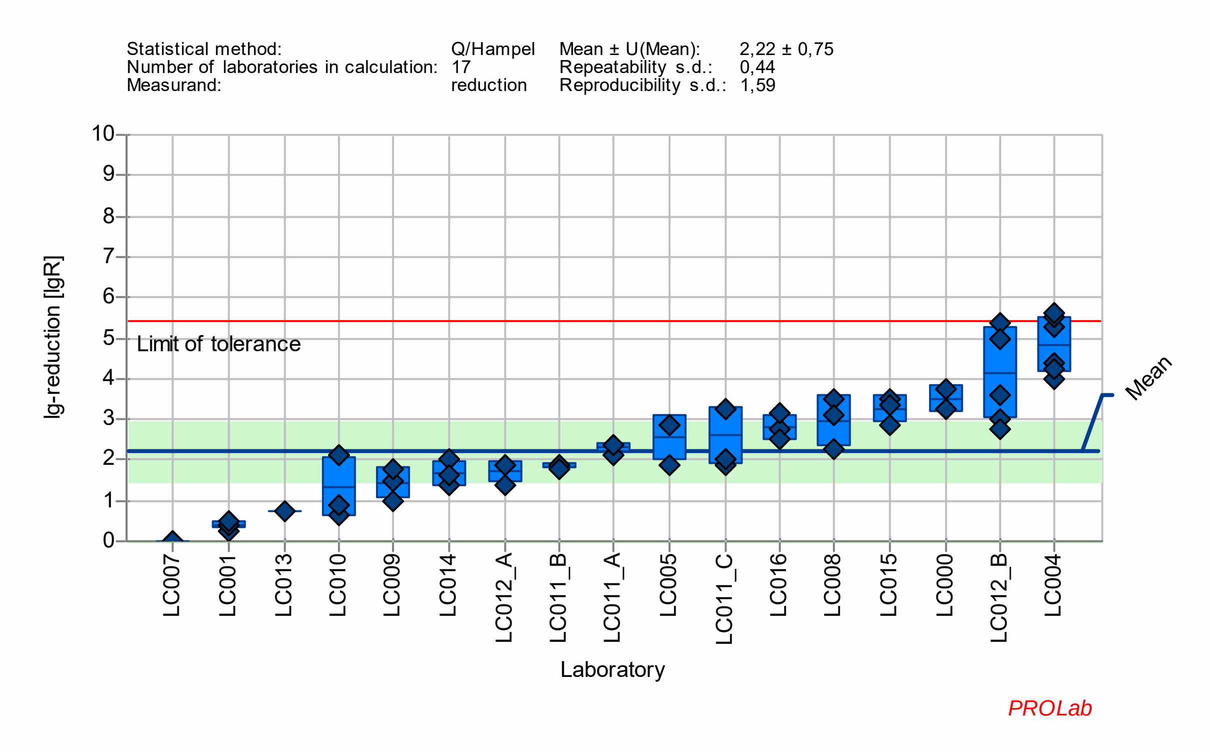

Figure 1: Mean and standard deviation of the lg-reduction for adenovirus with glutaraldehyde 50 ppm:30 min sorted by laboratory mean values.

Attachment to: Roesch KM, Hildebrandt C, Eggers M, Brill F, Cavalleri M, Gebel J, Hunsinger B, Loeffert S, Rausch M, Schwebke I, Steinhauer K, Exner M, Mutters NT. Enhancing the validity of virucidal activity testing of chemical disinfectants by establishing new reference substances according to EN 14476:2019. GMS Hyg Infect Control. 2025;20:Doc50. DOI: 10.3205/dgkh000579
